# Video Game-Based Electromyographic Biofeedback Interventions in Patients With Knee Osteoarthritis: A Clinical Trial

**DOI:** 10.7759/cureus.105993

**Published:** 2026-03-27

**Authors:** Mandeep Kaur, Eileen Krepkovich, James Patrie, Neal Richardson, Aaron Olowin, Amelia S Bruce Leicht, Xavier D Thompson, Susan Saliba, Joe Hart

**Affiliations:** 1 Physical Therapy, University of Texas Medical Branch at Galveston, Galveston, USA; 2 Engineering, Barron Associates Inc., Charlottesville, USA; 3 Public Health Services, University of Virginia School of Medicine, Charlottesville, USA; 4 Athletic Training and Clinical Nutrition, University of Kentucky, Lexington, USA; 5 Kinesiology, Louisiana State University, Baton Rouge, USA; 6 Kinesiology, University of Virginia, Charlottesville, USA; 7 Orthopedics, University of North Carolina at Chapel Hill, Chapel Hill, USA

**Keywords:** interventions, knee osteoarthritis, muscle strength, quality of life, video games

## Abstract

Introduction: Exercise is considered the most effective, non-drug treatment for reducing pain and improving movement in patients with knee osteoarthritis (OA). The current study aimed to compare the efficacy of incorporating a Knee Biofeedback Rehabilitation Game for Osteoarthritis Therapy (KneeBRIGHT) (Barron Associates, Inc., Charlottesville, VA, USA) device into a 10-week rehabilitation course in patients diagnosed with knee OA.

Methods: This clinical trial used a parallel, prospective, single-blind, randomized controlled design. Participants were randomly allocated to the KneeBRIGHT (n = 17) or the control (n = 17) group. Quadriceps muscle strength, perceived knee function using the Knee Injury and Osteoarthritis Outcome Score (KOOS) and the International Knee Documentation Committee (IKDC) scale, and balance were measured prior to and immediately following the rehabilitation program. The KneeBRIGHT group did exercises using the electromyography (EMG)-based video games, while the control group performed the same traditional exercises without the video games. All patients attended a combination of in-clinic and home-based interventions for 10 weeks. Participants were interviewed at the end of the study.

Results: Thirty-four patients participated in the study. The KneeBRIGHT group showed an improvement in KOOS quality of life (QOL) (p = 0.002), while the control group demonstrated improvements in KOOS symptoms (p < 0.001), pain (p = 0.009), activities of daily living (ADL; p = 0.005), KOOS sports (p = 0.002), and KOOS QOL (p = 0.016) compared to the baseline. No differences in quadriceps strength were found. The key themes from the interviews indicated that the KneeBRIGHT games are motivating and are an effective alternative to regular exercises.

Conclusions: Patients exhibited improvements in perceived knee function and exhibited increased quadriceps strength using KneeBRIGHT games compared to traditional rehabilitation exercises. Playing video games may help maintain patient motivation and can be done at home independently, effectively reinforcing the therapeutic effect of exercises.

## Introduction

Osteoarthritis (OA) is the leading cause of physical disability [1] and is responsible for an economic burden of over $300 billion annually, with over $100 billion attributed to lost wages as a result of disability in the United States [2]. By 2030, the number of adults affected by OA in the United States is expected to reach nearly 67 million (25% of the adult population); of those, 25 million are expected to have activity limitations due to the condition [3]. Knee OA is among the leading factors in reduced physical activity [4] and quality of life (QOL) [5].

OA is a chronic, progressive disease characterized by the subsequent worsening of symptoms, including pain, reduced strength, and reduced range of joint motion. In the long term, both conservative [6] and surgical management [7] strategies pose a great clinical and economic burden on the health system. Physical therapy, specifically exercise, remains the mainstay of treatment options. It is known that strength training can relieve pain, alleviate stiffness, enhance muscle strength, and improve physical function [8]. To maximize functional recovery, patients must adhere to long-term exercise routines. However, there is poor compliance with exercises among patients with knee OA [9]. Additionally, the repetitive nature of rehabilitation exercises can be a deterrent for many patients. These challenges indicate the strong need to provide interactive, engaging, and accessible options for physical therapy-based interventions.

The use of active video games has been investigated for their therapeutic utility in knee OA patients conducting muscle-strengthening rehabilitation programs [10]. Results indicated less pain and improved mobility among patients. However, that study used commercially available video games, which can be enjoyable to players but have no therapeutic value. These games encourage non-specific (and sometimes undesirable) movements, do not permit patient-specific settings, and can be frustrating for individuals with more severe impairments [11]. Electromyographic (EMG) biofeedback has been used for rehabilitation purposes in knee OA, patellofemoral pain, post-meniscectomy, and knee replacement [12]. Yet, despite this clinical utility, existing EMG biofeedback systems are largely utilitarian in design; they provide little patient engagement, offer minimal interactive feedback, and have not been optimized to support long-term adherence to home-based exercise programs. This represents a critical gap: the therapeutic precision of EMG biofeedback exists in isolation from the motivational qualities that drive sustained patient participation. We therefore propose that a purpose-built, game-based EMG-biofeedback system need not outperform conventional EMG-biofeedback in clinical outcomes such as pain and functional mobility, but rather demonstrate non-inferiority on these measures while achieving meaningful improvements in patient adherence, confidence, and usability. Providing real-time, game-mediated biofeedback may enable patients to gain conscious control over neuromuscular processes, improve movement accuracy, and remain engaged with rehabilitation over clinically meaningful timeframes [13].

We developed Knee Biofeedback Rehabilitation Game for Osteoarthritis Therapy (KneeBRIGHT) (Barron Associates, Inc., Charlottesville, VA, USA), a biofeedback-based game that enables patients to perform exercises targeted at optimal functional improvement for OA. A prior feasibility study successfully developed the KneeBRIGHT prototype, which combines muscle-sensing hardware and video game software, and integrated both into a virtual world experience that guided knee OA patients through knee rehabilitation exercises [14]. A preliminary feasibility study of the KneeBRIGHT system demonstrated that the game successfully achieved quadriceps muscle activation in a single rehabilitation session compared to a commercially available biofeedback unit [14].

The primary objective of this efficacy study was to evaluate whether a 10-week KneeBRIGHT would produce non-inferior outcomes to standard rehabilitation in patients with knee OA across three primary outcome domains: quadriceps muscle strength, balance, and patient-reported knee function as measured by the Knee Injury and Osteoarthritis Outcome Score (KOOS) and International Knee Documentation Committee (IKDC). It was therefore hypothesized that KneeBRIGHT would be non-inferior to standard rehabilitation in improving quadriceps strength, balance, and perceived knee function, including pain, ADL, sport and recreation, and knee-related QOL, over the 10-week intervention period. Secondary outcomes included comparing home exercise adherence and assessing the usability and acceptance of EMG biofeedback-driven video game exercises through qualitative analysis of semi-structured interviews.

This article was previously presented as a meeting abstract at the Combined Sections Meeting (February 3, 2022) and as proceedings at the Journal of Orthopaedic & Sports Physical Therapy (52(1):p CSM96-CSM97, January 2022).

## Materials and methods

Study design

This was a small phase II prospective, parallel, single-blind, randomized, and controlled clinical trial. We recruited patients aged 35-75 years with a current physician-diagnosed knee OA, confirmed through medical and radiographic record reviews, who were referred for physical therapy. Exclusion criteria included participants with diagnosed balance or vestibular disorders, peripheral neuropathy, skin conditions that prevent EMG electrode placement, cognitive deficits resulting in impairment of the ability to follow instructions or complete any part of the study, prisoners, and individuals who were pregnant (by self-report), non-English-speaking, and visually and/or hearing impaired. The study was approved by the University of Virginia Institutional Review Board, and all participants provided informed consent. The study was registered at ClinicalTrials.gov (NCT04187092).

Procedures

After screening, enrollment, and blinded baseline evaluations, subjects were randomized to either the standard rehabilitation group or the KneeBRIGHT group in a 1:1 ratio using a random number generator. Block randomization was done by JH, a senior investigator, on the team. Numbers generated were concealed in an envelope. MK enrolled the participants and assigned them to either group based on the assigned number. It was determined to have at least 22 participants per group (accounting for dropouts from the 50 recruited subjects), to achieve statistical power of 80% to detect a change of 13 points, which is reasonable given that this value is lower than the minimal detectable change in Knee osteoarthritis outcome scale scores for knee OA patients (this value ranges from 13.4 for the pain subscale to 21.1 for the quality of life subscale). Participants completed a 10-week intervention, after which another blinded evaluation was performed. Additional surveys and semi-structured interviews were also conducted for qualitative analysis. Following the 10-week study period, participants were offered the option to continue for an additional two weeks of rehabilitation if they wanted. All the data were collected at the University of Virginia.

Rehabilitation intervention

Participants in both groups completed a structured 10-week exercise program comprising three sessions per week, for a total of 30 sessions. Sessions were delivered across a combination of settings: 8 visits were conducted in-clinic, while 22 were home-based, of which 10 were supervised via video conferencing and 12 were unsupervised. Each session lasted approximately one hour, ensuring a consistent intervention dose across both groups.

The transition from supervised to independent practice was structured deliberately to maximize familiarity with the exercise routines and equipment before reducing oversight. The program began with two weekly in-clinic visits, with home-based sessions introduced gradually as patients demonstrated competence and confidence. This progressive shift in supervision is detailed in Table 1. Participants were also offered the option to extend their program by an additional two weeks, with continued reporting to the study team, providing a standardized pathway for those requiring a longer consolidation period.

**Table 1:** Overview of the exercise sessions, showing a weekly schedule for patients completing three sessions of exercises weekly, both in clinic and at home.

	One-hour rehabilitation exercise sessions		
	In clinic, supervised	At home, supervised	At home, unsupervised
Week 1	2	1	0
Week 2	1	2	0
Week 3	1	1	1
Week 4	1	1	1
Week 5	0	2	1
Week 6	1	1	1
Week 7	1	0	2
Week 8	0	1	2
Week 9	1	0	2
Week 10	0	1	2
Optional week 11	0	0	3
Optional week 12	0	0	3

Both groups followed the same underlying progressive rehabilitation protocol, with session content adjusted based on ongoing symptom monitoring, including pain levels and functional tolerance, to allow each participant to incrementally increase their exercise volume over time. To support replication, progression was guided by the pre-planned protocol. Where a participant demonstrated difficulties during exercises or other risk factors, the exercise regimen was modified using a structured decision framework applied consistently across both groups. Modifications, determined by the supervising physical therapist, included one or more of the following: elimination of step-ups, which carry the highest balance demand; substitution of squats with wall-slides; or partial or full modification of squat exercises. In the KneeBRIGHT group, these adaptations were implemented directly through software settings, while control group participants received a modified instructional handout reflecting the same changes (Appendix 1). Participants in the KneeBRIGHT group were additionally provided with EMG-biofeedback sensors and a study laptop preloaded with the video game software for the full duration of the trial, ensuring consistent access to the intervention technology throughout all home-based sessions.

Standard knee rehabilitation group

The standard of care group used standard physical therapy equipment (towels, steps, elastic bands, etc.) while conducting exercises during both clinic and at-home sessions. Subjects were evaluated during all clinic sessions. Any changes in patient status or comfort with home exercise were noted, and the corresponding exercise regimen was modified as necessary.

Video game exercise intervention group

**Figure 1:**
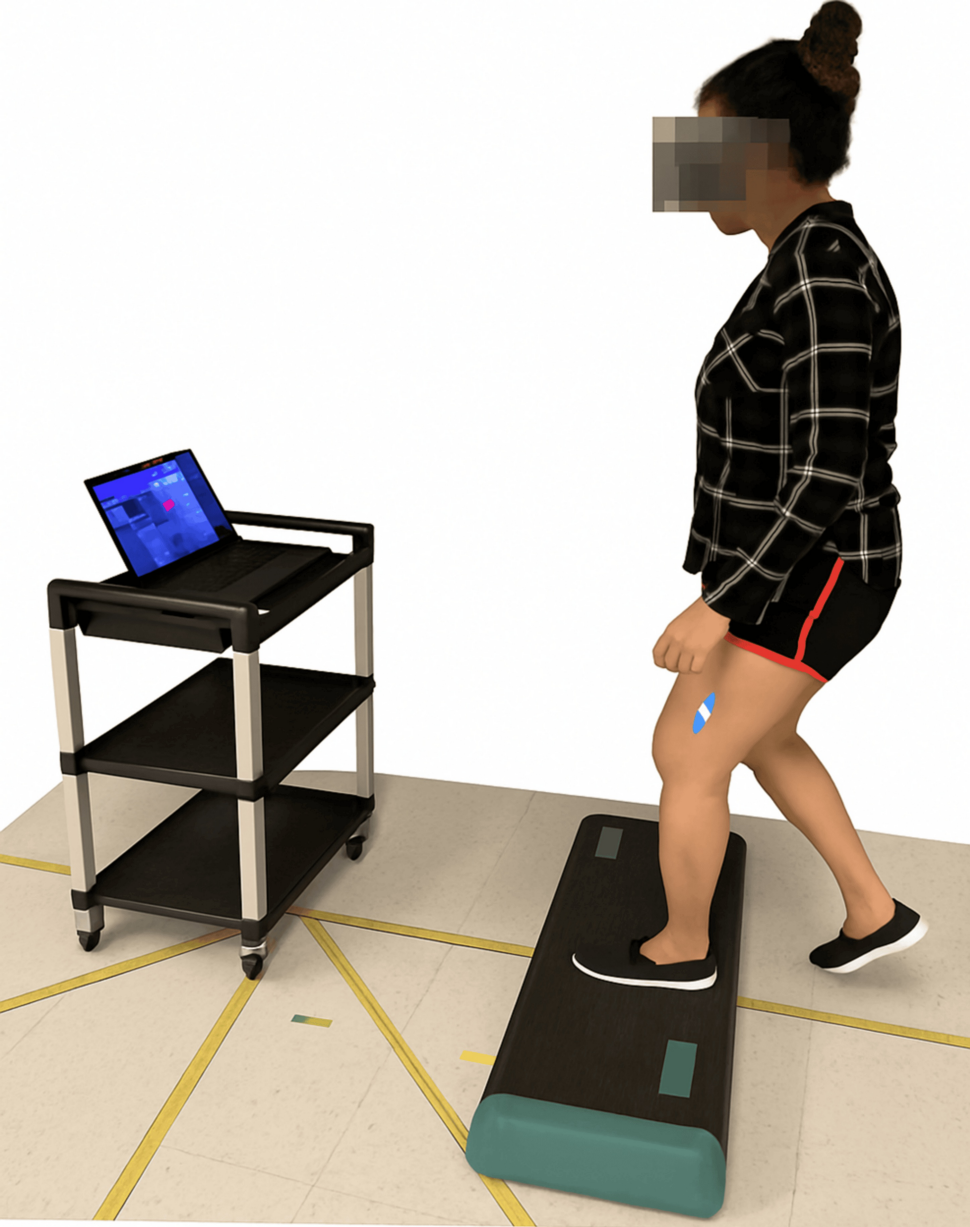
Patient positioning and electrode placement using the KneeBRIGHT system (Barron Associates, Inc., Charlottesville, VA, USA). KneeBRIGHT: Knee Biofeedback Rehabilitation Game for Osteoarthritis Therapy.

Participants randomized to the KneeBRIGHT group received instructions on applying the EMG biofeedback sensors and using the KneeBRIGHT video game. Surface electrodes were placed on the left and right vastus lateralis muscles, using custom-fabricated EMG sensors that wirelessly interfaced with the KneeBRIGHT video game software (Figure 1). Participants received feedback via a video game interface that included audible cues and rewards such as points and gold coins. Target contraction EMG-thresholds were computed in the game through a calibration procedure in which the player performed a maximum voluntary isometric contraction (MVIC) bilaterally. Game controls were modulated based on the intended percent MVIC. For example, lower-intensity exercises required a lower percentage of EMG activation threshold to manipulate the digital game vehicles, and higher-intensity exercises required a higher EMG activation based on calibration performed at each session. Exercises were focused on quadriceps muscle strengthening. Difficulty advancement was triggered when a participant reached the target muscle activation threshold across a defined number of consecutive successful repetitions, and the physical therapist retained the authority to override progression based on symptom monitoring. The exercise progressions were monitored and adapted by maintaining targeted quadriceps activation through the games and included tasks of weight shifting, single leg balance, mini-squats, and knee extension. Successful gameplay earned experience points that translated to increases in challenges and complexity of the tasks.

**Figure 2:**
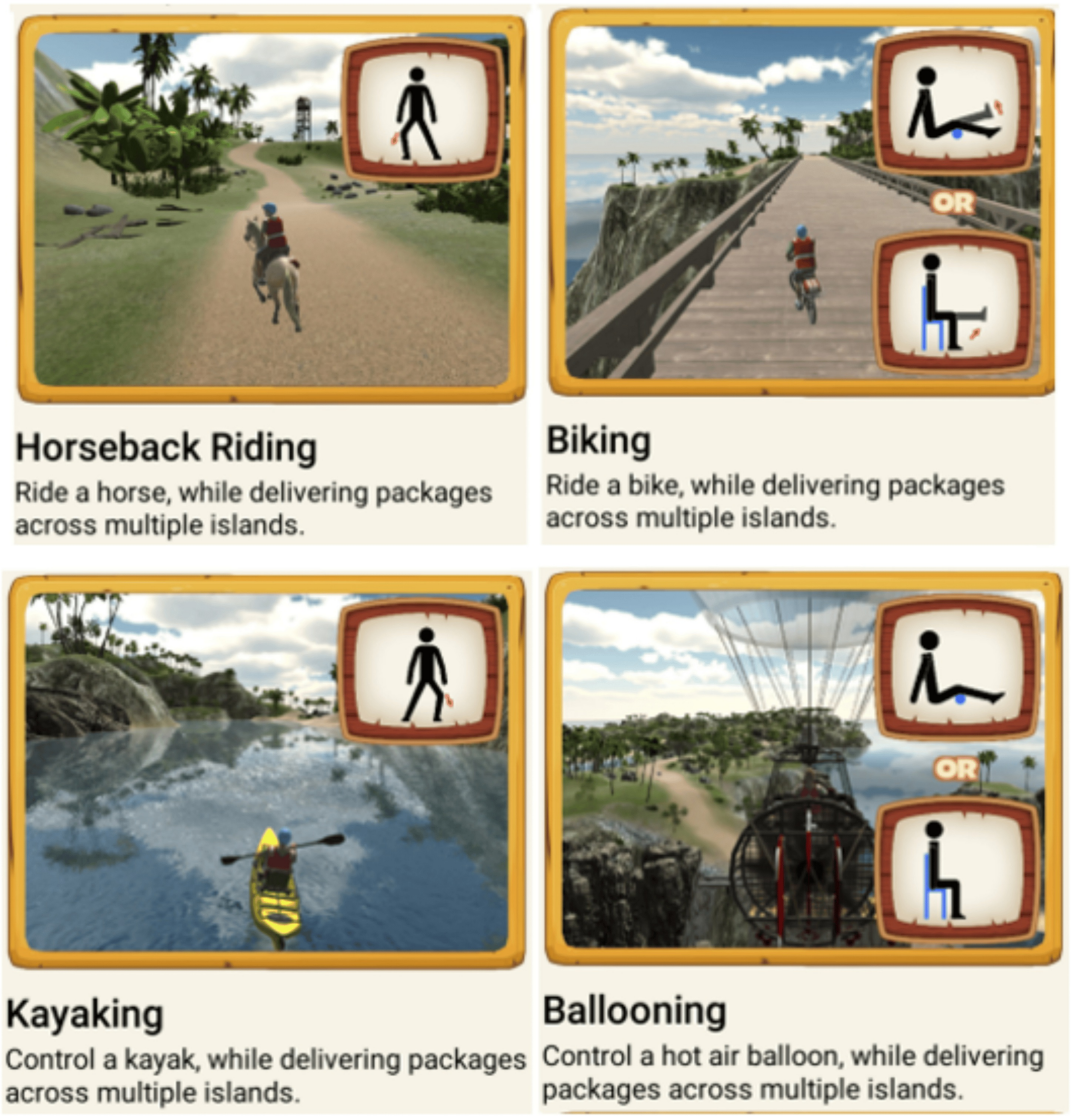
Video game-based interventions: game interfaces and associated exercises. Note: Permission to publish screenshots of the game interface was obtained from Barron Associates Inc. and the University of North Carolina.

The video game was developed based on feedback from the targeted age group so that it would be engaging, understandable, and competitive for the intended population. The game's tasks and storyline involved delivering supplies around a tropical island through various game levels. Each level had a different delivery vehicle, including a kayak, hot air balloon, horseback, and bicycle (Figure 2).

Outcome assessments

Physical and self-reported outcomes were collected by blinded evaluators both prior to and immediately following the 10-week intervention. No additional assessment was performed if participants opted for additional exercise sessions past the 10-week intervention period.

Patient-reported outcomes

The KOOS measures patients’ opinions about their knee and associated problems, such as ligament injuries [15]. It has five domains: (1) pain frequency and severity during functional activities; (2) symptoms such as the severity of knee stiffness and the presence of swelling, grinding or clicking, catching, and range of motion restriction; (3) difficulty experienced during activities of daily living (ADL); (4) difficulty experienced with sport and recreational activities; and 5) knee-related QOL. Scoring involves a range from 0% to 100%, where 0 = extreme problems and 100 = no problems. Higher values indicate better knee function. The minimal clinically important difference (MCID) for the KOOS varies by subscale: 8-10 points for pain, 8-10 points for symptoms, 8-10 points for ADL, 12-15 points for sports and recreation, and 12-15 points for QOL, as established in knee OA and post-surgical populations [16]. The IKDC subjective form was also used to measure regional knee health in terms of knee function and is reliable and valid for knee ligament injuries. Scores range from 0 to 100, where 100 = no limitation with daily or sporting activities and the absence of symptoms [17]. The MCID for the IKDC has been reported at approximately 11.5 points in patients with knee pathology, including osteoarthritis and ligament injuries [18].

Thigh muscle strength testing 

Isokinetic, concentric knee extension strength was measured bilaterally with a multimode dynamometer (System 4, Biodex Medical Systems, Shirley, NY, USA) at speeds of 90°/sec and 180°/sec. The participants completed practice trials on each limb for practice and familiarization until they were confident. The less affected side was tested first. The participants provided maximal effort through their full range of motion for eight trials. All strength data were presented as torque normalized to body mass (nm/kg).

Balance testing

Balance was assessed as an exploratory outcome. Balance testing was performed on Tekscan MobileMat (Tekscan, Inc., Norwood, MA, USA) and processed by FootMat software (Tekscan, Inc., Norwood, MA, USA). Patients stand on a single leg with the knee in approximately 30° knee flexion on a pressure mat for 30 seconds, while balance measures were measured as centimeters per second (cm/s). Subjects performed two repetitions on both the right and left sides. Bilateral balance was also tested with the patient instructed to perform the bilateral squat. Subjects were instructed to hold the squat for 30 seconds, and two repetitions were performed. Testing was performed by a blinded investigator with experience and training to perform these measures in patients with knee injuries. All participants first completed each trial on their less involved limb to allow familiarization with the task prior to completion on the involved limb.

Additional surveys and semi-structured interviews

At the post-rehabilitation evaluation only, participants in the KneeBRIGHT group completed the Technology Acceptance Model (TAM) questionnaire [19,20] to assess participants’ levels of engagement and technology acceptance, and the System Usability Score (SUS) assessment. The TAM instrument is a four-point Likert scale (1 = strongly disagree; 4 = strongly agree) focused on the key categories that are the most predictive of acceptance and usability in medical and gaming technology: perceived usefulness, attitude towards using, self-efficacy, perceived efficacy, and perceived ease-of-use. The maximum score is 5; a mean score greater than 3 is considered positive for technology acceptance.

The SUS is an additional Likert-scale tool that is commonly used to evaluate usability [21]. The SUS was used to evaluate the usability of the KneeBRIGHT system. It consists of a 10-item questionnaire with five response options for respondents, from strongly agree to strongly disagree [21]. It is scored from 0 to 100; based on normative data, a SUS score above 68 would be considered above average, and anything below 68 is below average [22].

At the end of the study, individual, face-to-face structured interviews were also conducted. The research team developed and refined the interview guide, which had open-ended questions (Table 2).

**Table 2:** Interview questions used for the KneeBRIGHT and standard rehabilitation groups. KneeBRIGHT: Knee Biofeedback Rehabilitation Game for Osteoarthritis Therapy.

Questions for patients randomized to rehabilitation with the KneeBRIGHT video game	Questions for patients randomized to standard rehabilitation
Have you ever played video games or games on a computer? If yes: What games did you play? How often did you play? Can you describe what you liked about the game(s)? What didn’t you like about the game(s)?	Could you describe your overall experience with the exercises you performed over the last few weeks?
What are your thoughts about using computer games as part of an exercise program at home?	What are your thoughts about the physical challenge of the exercises?
What are your thoughts about playing exercise games in a group setting?	What was the most positive aspect of performing the exercises?
What are your thoughts about playing exercise games with others over the Internet?	Are there specific aspects of the exercises that you liked?
Are there specific aspects of exercises you liked or did not like, and why?	What is the most negative aspect of performing the exercises?
If you could make one change to the exercise routine or sessions, what would it be?	If you could make one change to the exercise routine, what would it be?
	Is there anything else you’d like to say about the experience?

Statistical analysis

Muscle strength, KOOS, and IKDC scores were compared between the standard rehabilitation group and the KneeBRIGHT group by way of analysis of covariance (ANCOVA) using the baseline scores as the model covariate. We compared balance performance between treatment groups using a 2X2 ANOVA with paired t-tests as post hoc comparisons. Optional exercise duration after the initial 10-week period was compared using t-tests. A p ≤ 0.05 decision rule was used as the criterion for rejecting the null hypothesis that the standard rehabilitation group would have greater functional improvements and exercise duration, compared to the KneeBRIGHT group. 

Qualitative data were analyzed using a Microsoft Excel 2019 spreadsheet (Microsoft Corp., Redmond, WA, USA). A general inductive method was used to summarize the interview data [23]. In this method, the transcriptions were read multiple times, and a researcher identified and coded text segments reflecting the participants’ experiences. The codes were categorized and the researchers developed links between these categories and identified the themes relevant to the research aims. The categories, emerging themes, and sub-themes were discussed and confirmed by the research team.

## Results

**Figure 3:**
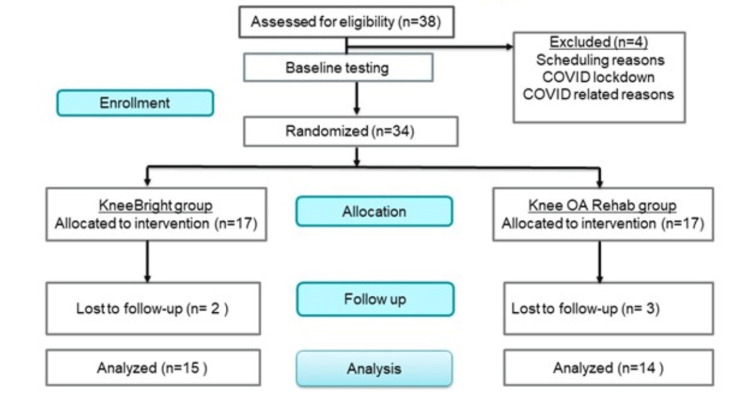
Allocation and randomization of the participants. KneeBRIGHT: Knee Biofeedback Rehabilitation Game for Osteoarthritis Therapy, OA: osteoarthritis.

We screened thirty-eight patients diagnosed with knee OA between December 2019 and May 2021. Thirty-four patients were enrolled and randomized into either the control or KneeBRIGHT groups. The average age of participants was 63.4 ± 6.5 (range 53.8-74.7) years for the standard rehabilitation group and 69.3 ± 4.9 (range 59.8-75.9) years for the KneeBRIGHT group. Women made up a greater percentage of subjects in the standard rehabilitation group than in the KneeBRIGHT group (p = 0.08), and on average, participants in the standard rehabilitation group were younger than those in the KneeBRIGHT group (p = 0.011). Due to the COVID-19 lockdown, final strength testing of some of the participants was not completed; therefore, four participants from the knee bright group and two from the standard rehabilitation group were excluded from strength analysis (KneeBRIGHT: n = 11; standard rehab: n = 12) (Figure 3).

**Figure 4:**
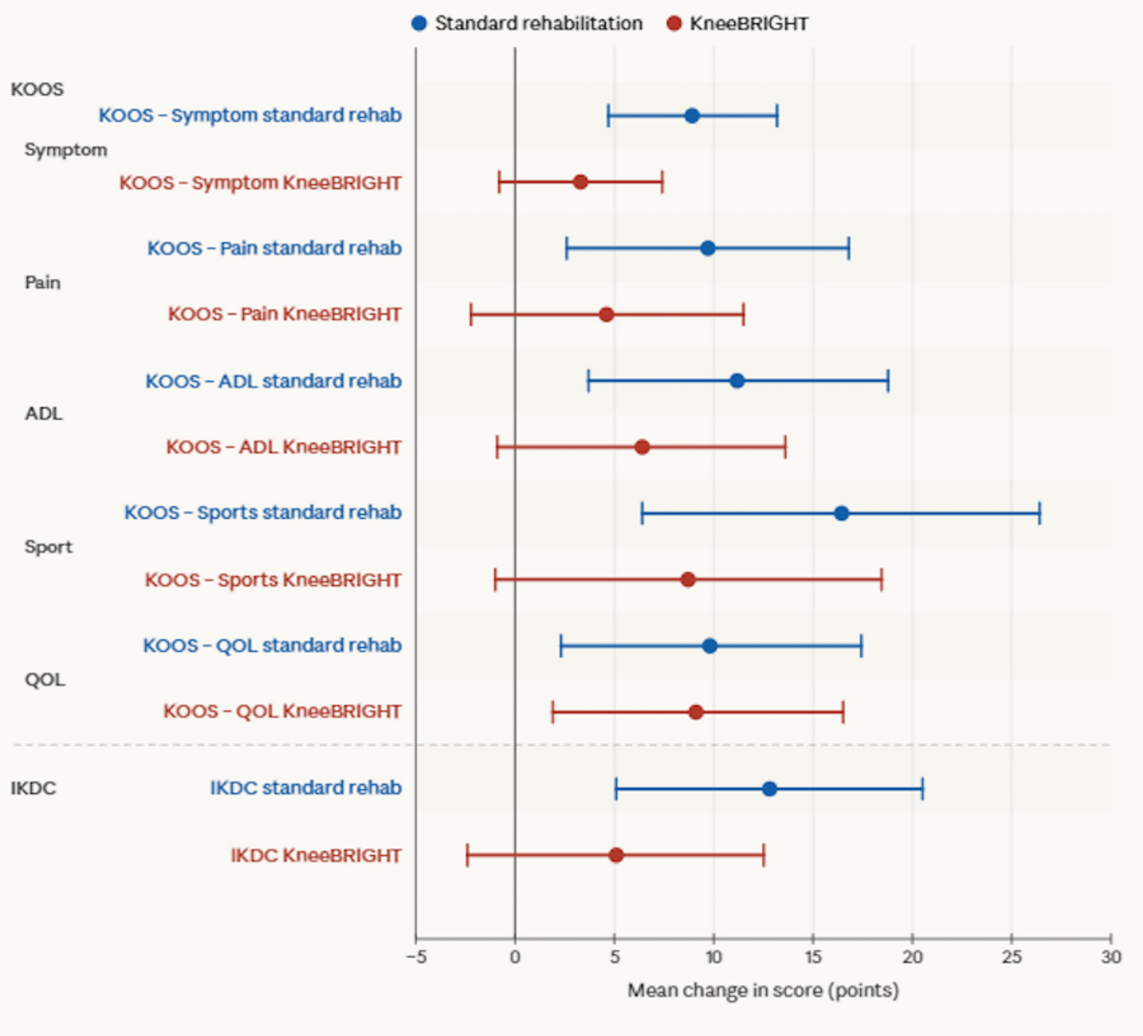
Mean change among Knee Injury and Osteoarthritis Outcome Score (KOOS) and International Knee Documentation Committee (IKDC) following the intervention. Error bars represent the confidence intervals for the mean score change; point estimates represent the mean score change. ADL: activities of daily life, QOL: quality of life.

The standard rehabilitation group had a significant improvement in scores for KOOS symptoms (p < 0.001), pain (p = 0.009), ADL (p = 0.005), sports (p = 0.002), and QOL (p = 0.016) at 10 weeks compared to the baseline scores (Figure 4).

The KneeBRIGHT group showed significant improvements in KOOS QOL scores compared with the baseline (p = 0.002). Within-group changes were evaluated against established MCID thresholds to determine the clinical meaningfulness of observed improvements. For the KOOS, the standard rehab group achieved clinically meaningful within-group improvements across four of five subscales, exceeding the MCID threshold for symptom (+8.93 points; MCID: 8-10), pain (+9.72 points; MCID: 8-10), ADL (+11.24 points; MCID: 8-10), and sports and recreation (+16.46 points; MCID: 12-15), with QOL falling marginally below the threshold (+9.82 points; MCID: 12-15). In contrast, the KneeBRIGHT group did not reach the MCID threshold on any KOOS subscale, with changes ranging from +3.29 points (symptom) to +9.17 points (QOL).

For IKDC scores, the standard rehab group showed significant improvement at the end of 10 weeks of intervention compared with baseline (p = 0.002), whereas the KneeBRIGHT group showed no significant improvement (Figure 4). Regarding within-group changes, IKDC scores revealed that the standard rehab group achieved a clinically meaningful improvement of +12.86 points, exceeding the established MCID of 11.5 points, whereas the KneeBRIGHT group's improvement of +5.06 points fell below this threshold. These within-group findings should be interpreted cautiously, however, as between-group comparisons using covariate-adjusted ANCOVA revealed no statistically significant differences in the magnitude of change between the KneeBRIGHT and standard rehab groups across any KOOS subscale or IKDC score (Table 3). 

**Table 3:** Means and standard deviations for the analyses of the KOOS, IKDC, extension normalized peak, and normalized average torque (nm/kg). *No significant differences were found in the baseline scores between the two groups at p < 0.05. p-values reported are for the covariate-adjusted ANCOVA. ADL: activities of daily living, QOL: quality of life, KneeBRIGHT: Knee Biofeedback Rehabilitation Game for Osteoarthritis Therapy, KOOS: Knee Injury and Osteoarthritis Outcome Score, IKDC: International Knee Documentation Committee, ANCOVA: analysis of covariance.

				Score change (12-week baseline)			
	Group	Pre (SD)	Post (SD)	Mean D	95% CI	p-value	Covariate-adjusted p-value
KOOS*							
Symptom	Standard rehab	58.67 (17.0)	67.60 (16.7)	8.9	(4.7, 13.2)	<0.001	0.138
	KneeBRIGHT	60.71 (15.9)	64.0 (15.7)	3.3	(-0.8, 7.4)	0.106	
Pain	Standard rehab	63.89 (18.6)	73.61 (19.9)	9.7	(2.6, 16.8)	0.009	0.157
	KneeBRIGHT	65.93 (9.0)	70.6 (12.10)	4.6	(-2.2, 11.5)	0.177	
ADL	Standard rehab	71.85 (18.0)	83.09 (16.6)	11.2	(3.7, 18.8)	0.005	0.120
	KneeBRIGHT	71.76 (15.5)	78.14 (13.7)	6.4	(-0.9, 13.6)	0.083	
Sport	Standard rehab	37.14 (34.3)	53.6 (30.8)	16.4	(6.4, 26.4)	0.002	0.166
	KneeBRIGHT	36.6 (25.0)	45.33 (27.6)	8.7	(-1.0, 18.4)	0.078	
QOL	Standard rehab	44.20 (22.5)	54.02 (23.7)	9.8	(2.3, 17.4)	0.013	0.256
	KneeBRIGHT	40.0 (18.4)	49.17 (15.3)	9.1	(1.9, 16.5)	0.016	
IKDC*							
IKDC	Standard rehab	48.44 (20.3)	61.30 (21.3)	12.8	(5.1, 20.5)	0.002	0.084
	KneeBRIGHT	48.74 (15.9)	53.80 (16.0)	5.1	(-2.4, 12.5)	0.173	
Muscle strength (nm/kg) 90°/sec*							
Peak torque*	Standard rehab	0.90 (0.41)	1.01 (0.49)	0.11	(-0.02, 0.23)	0.082	0.948
	KneeBRIGHT	0.94 (0.49)	1.04 (0.49)	0.11	(-0.02, 0.24)	0.099	
Average torque*	Standard rehab	0.80 (0.37)	0.90 (0.46)	0.10	(-0.01, 0.22)	0.082	0.824
	KneeBRIGHT	0.84 (0.44)	0.93 (0.47)	0.10	(-0.02, 0.22)	0.113	
180°/sec*							
Peak torque*	Standard rehab	0.89 (0.62)	0.83 (0.34)	-0.06	(-0.25, 0.13)	0.514	0.346
	KneeBRIGHT	0.74 (0.30)	0.81 (0.32)	0.07	(-0.13, 0.27)	0.499	
Average torque*	Standard rehab	0.69 (0.29)	0.72 (0.29)	0.03	(-0.08, 0.14)	0.547	0.762
	KneeBRIGHT	0.68 (0.29)	0.68 (0.28)	0.00	(-0.19, 0.18)	0.971	0.346

**Figure 5:**
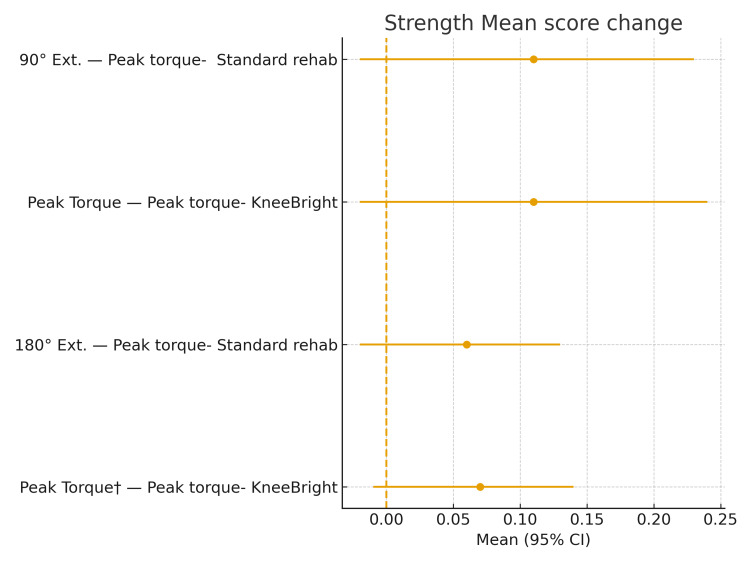
Mean change for muscle strength following the intervention measured at two isokinetic velocities: 90°/sec and 180°/sec. Error bars represent the 95% confidence intervals for the mean change; point estimates represent the mean score change. KneeBRIGHT: Knee Biofeedback Rehabilitation Game for Osteoarthritis Therapy.

Both groups had no significant differences in baseline scores for peak and average quadriceps torque at 90°/sec and 180°/sec for quadriceps strength (Table 3). There were no significant differences in pre-test/post-test strength outcomes (Figure 5). No significant differences were found for the change in the quadriceps peak and average torque at 90°/sec and 180°/sec for both groups with the covariate-adjusted mean of the distribution for the 10-week intervention (Table 3). 

Only participants in the KneeBRIGHT group opted to extend their exercises for an additional two weeks beyond the required 10-week regimen. The KneeBRIGHT group had higher adherence to home exercise sessions than the standard rehab group (KneeBRIGHT = 84% vs. standard rehab group = 70%; p = 0.001). This provides strong evidence that patients doing home-based exercise are more likely to continue exercise sessions when conducted with the KneeBRIGHT system than they would be doing standard home PT-prescribed exercise.

No significant differences were found between groups for the single-leg balance and bilateral balance performance assessments (Table 4).

**Table 4:** Mean (standard deviations) for balance measures (COP excursions) expressed as average velocity across the entire balance trial (cm/s) among the pre- and post-intervention groups for the involved limb. The effect size (Cohen’s d) represents the difference in the pre-post-change between groups in single leg and bilateral balance outcome variables. KneeBRIGHT: Knee Biofeedback Rehabilitation Game for Osteoarthritis Therapy.

	KneeBRIGHT group		Rehab group		Effect size (95% CI)	Main effect	Interaction effect
	Pre	Post	Pre	Post			
Single leg balance	103.3 (44.8)	105.4 (25.1)	89.7 (20.8)	93.0 (21.5)	0.53 (-0.23 to 1.25)	0.647	0.915
Bilateral balance	32.5 (3.4)	37.0 (8.0)	35.4 (13.9)	38.26 (10.6)	-0.13 (-0.86 to 0.60)	0.235	0.787

Survey scores

A composite score was obtained by summing the Likert responses for all categories. The mean KneeBRIGHT TAM responses were found to be 3.2 out of 4, indicating strong technology acceptance. Based on the SUS results (a mean score of 67.5 (SD = 27)), the results indicate average system usability.

Qualitative analysis of the interviews

Two independent coders (MK and EK) conducted primary coding and agreed on three key themes. Three key themes emerged from the qualitative analysis of the interview transcriptions (Appendix 2) and are discussed below.

Key Theme 1: The KneeBRIGHT Games for Exercise Are Motivating Because They Are Fun and Keep You Accountable

Participants mentioned that KneeBRIGHT games used in this study were engaging because of the fun elements, such as the travel visuals (quotes 1, 6; P1, P35), and that playing games to exercise was more fun than typical physical therapy (quotes 2, 3, 8; P7, P25, P28). For some participants, the game exercises were engaging and held their attention even with distractions (quotes 1, 4; P1, P25). Two participants mentioned that they particularly appreciated that the games provided a structured approach to exercises where they do not have to focus on counting the number of repetitions (quotes 1, 3, 8; P1, P25, P28). Several participants mentioned how real-time feedback was helpful to them during the session for keeping them accountable (quotes 7, 8; P28) and motivating them to work harder (quotes 9, 10, 11; P28, P31, P37).

Key Theme 2: The Exercises Conducted With the KneeBRIGHT Games Are an Effective Alternative to Regular Exercise

Several participants mentioned that they feel that games made them stronger (quotes 12, 14, 15, 16; P1, P14, P25, P26). When comparing the game exercise to traditional physical therapy exercise, participants noted that the actual exercises were similar (quote 13; P7). Furthermore, they appreciated the additional structure from the games (quote 19; P21) and that doing exercise with the game at home is convenient because they do not have to drive anywhere (quote 18; P14).

Key Theme 3: Patients' Thoughts on the Hardware and Software Usability of the System

Hardware and software usability of the system: The following subthemes were found regarding system usability. Patients would like additional encouragement regarding their performance and progress. One participant mentioned the need for feedback regarding their form when performing exercises during the game (quote 20; P4), preferring the ability to get feedback from a physical therapist to correct or verify the mechanics of exercises during the game (quote 23; P4). Several participants mentioned that they would like to see a summary of their progress at the end of the session (quote 20; P4), and specifically get more updates on the progression of their knee health (quotes 21, 22, 23; P26, P6).

Room for improvement in hardware and software: Participants expressed frustration with their inability to finish an exercise due to hardware issues (quote 24; P1), along with overall problems with transmitters (quote 25; P21). Regarding the game software, several participants mentioned struggling with the timing of different events in the game, noting short transition times (quote 27, 29; P7, P21) and inconsistency with how quickly or slowly certain targets would move in the game (quote 28; P26). Two participants mentioned how sometimes they would miss locating the targets in the game as those were hidden under other items (quotes 26, 28; P6, P26). Two participants also emphasized improving he consistency of the sensitivity of the game control so they could handle the games better (quotes 28, 30; P26, P35).

## Discussion

The primary aim of this study was to compare functional outcomes (pain, strength, and movement quality, assessed via KOOS score and peak knee extension torque) among the KneeBRIGHT and standard rehabilitation groups, following a 10-week intervention. To the best of our knowledge, this is the first study to compare the therapeutic effects of playing EMG-based active video games with standard rehabilitation exercises in patients with knee OA. Patients in the standard rehabilitation group had statistically significant improvement in all KOOS change scores subscales, namely, symptoms, pain, sports, ADL, and QOL, while KneeBRIGHT patients perceived statistically significant change in the QOL at the end of the intervention. The standard rehab group achieved clinically meaningful within-group improvements across four of the five KOOS subscales, with only QOL falling below the MCID threshold. The KneeBRIGHT group, by contrast, did not reach the MCID threshold on any subscale, suggesting that while participants in both groups showed positive trends, the magnitude of improvement in the KneeBRIGHT group was consistently sub-threshold. The relatively smaller sample size and short intervention window may have limited the KneeBRIGHT group's ability to accumulate sufficient training volume to produce MCID-level gains, which is an important consideration for future trial design. For IKDC scores, both groups entered the trial with comparable baseline IKDC scores (48.44 vs. 48.74), confirming good pre-intervention equivalence. However, their trajectories diverged meaningfully over the course of the intervention. The standard rehab group crossed the MCID threshold with a +12.86 point improvement, while the KneeBRIGHT group's +5.06 point gain remained substantially below it, indicating that the standard rehabilitation group perceived better knee function at the end of intervention, while no significant change in mean scores was reported in the KneeBRIGHT group. Significant improvements in KOOS and IKDC scores in standard rehabilitation are no surprise. The efficacy of physical therapy in reducing pain and knee symptoms is well established [24]. Significant differences in the KOOS QOL scores in the KneeBRIGHT group indicate the system's efficacy in improving knee health and overall QOL.

It should be noted that participants in the KneeBRIGHT group are significantly older than those in the standard rehabilitation group (p = 0.011). Although randomization was employed, this difference warrants consideration when interpreting between-group outcome differences, as age is an independent determinant of multiple physiological and behavioral factors directly relevant to the outcomes measured in this trial [25]. Age-related muscle loss and reduced balance are well-documented among older adults. Older participants may therefore have entered the trial with a reduced capacity for strength and balance adaptation over a 10-week period, as the anabolic response to resistance exercise diminishes with advancing age. This may partly explain the absence of significant quadriceps strength and balance gains in the KneeBRIGHT group, independent of the intervention itself. Also, older adults are known to demonstrate initial reluctance toward digital technologies compared to younger adults [26]. While qualitative interview data indicated that participants found the KneeBRIGHT games motivating, it is possible that older participants required a longer familiarization period than the 10-week window afforded.

The adherence to exercise was higher in the KneeBRIGHT group compared to the standard rehab group. A plausible explanation is that the game interface is fun, engaging, and motivating. KneeBRIGHT seeks to maximize the potential for adherence improvements via biofeedback through EMG, which ensures that exercises are performed correctly and optimized for patient ability level; also, the ability to do the exercises at home through the system, like the one used in this study, promotes adherence. This is also apparent from the qualitative findings where participants reported that real-time EMG-biofeedback kept them accountable during exercises, that gameplay sustained their attention even in the presence of distractions, and that the game structure removed the cognitive burden of counting repetitions, all of which are directly relevant to maintaining consistent exercise participation during unsupervised home sessions. Acceptance of technology was indicated from the TAM scores in this study (3.2 out of 4.0), which indicates good acceptance; similarly, SUS scores were on average. Incorporating adherence monitoring features can provide clinicians with the ability to see the patient's metrics of exercise. Maintaining therapeutic exercise is critical in the treatment of knee OA [27]. In the present study, playing active video games could maintain therapeutic effects for 10 weeks. KneeBRIGHT exemplifies a new paradigm of in-home rehabilitation for knee OA by biofeedback, presentation of exercise performance, and adherence data. This system can log exercise performance. These quantitative metrics go well beyond the self-reported questionnaires that are the only current means of exercise adherence assessment used in clinical practice.

The primary outcome from this study demonstrates no significant differences in strength outcomes between the two treatment groups, meaning that, on average, the strength gains after completion of the rehabilitation program were no different in patients who performed standard rehabilitation and patients who performed rehabilitation with KneeBRIGHT. This finding demonstrates that the use of EMG-biofeedback video game-based rehabilitation resulted in strength gains that were no different than standard physical therapy practice. Our previous study [28] compared a prototype KneeBRIGHT system with commercially available (non-video game-based) EMG-biofeedback among patients with knee OA. Patients in the KneeBRIGHT group generated 25% higher peak knee extension torque during a single session compared to the standard rehab sessions. However, in the present study, no statistically significant differences in strength were observed. This was surprising, given the results from the prior phase. The results from our current study may have been impacted by the duration of our rehabilitation protocol (10 weeks) and the general heterogeneity in the OA disease state among the patients included. Specifically, participants in the current study (compared to the prior phase study [14]) were at different stages of knee OA severity and had varied levels of pain. Higher pain could interfere with the exercise participation levels [29] and may be an area of consideration in designing future rehabilitation clinical trials in patients with knee OA. Studies with a longer follow-up period are needed. The effects of active video games on outcomes among a variety of rehabilitation populations demonstrated improvement in physical health [10].

The current study also demonstrated no statistically significant improvements or differences between groups in single leg or bilateral balance. The exercises incorporated in the protocol of control or the KneeBRIGHT group were primarily designed to improve muscle strength through higher activation. For balance improvement, different types of exercises would be required [30]. In the present study, active video games required the participants to see a target on the screen and dynamically interact with it. Their target for contracting the muscles was based on the EMG calibration performed at the beginning of the study, which was dependent on a maximal muscle contraction during sitting or standing. Given the nature of the game calibration and the modes of exercises incorporated into the rehab programs, it is not surprising that patients did not exhibit balance improvements. Future applications of video game therapy may incorporate different exercises and matching video game interactions that specifically target different musculoskeletal and performance outcomes.

There is poor compliance with the exercises in patients with knee OA. Therefore, with technological advancements, especially in the field of rehabilitation, newer ways for engaging patients are being explored. A recent narrative review focused on the patient perspectives of use of digital health for intervention delivery in OA-focused care found digital health to be as effective as traditional treatments for patient education and exercise interventions. Importantly, this positions digital tools such as KneeBRIGHT not as replacements for conventional rehabilitation, but as adherence-enhancing adjuncts, designed to deliver the same evidence-based therapeutic content while embedding it within an engaging, game-mediated environment that sustains patient motivation across both supervised and unsupervised settings. This adjunctive role is particularly relevant in the home-based context, where the absence of clinical supervision makes intrinsic motivation the primary driver of exercise adherence. Patients in the current study indicated that videogame-based exercises were motivating, fun, and competitive, as reflected in qualitative "Theme 1," supporting the premise that KneeBRIGHT can complement rather than replace standard care by addressing the motivational gap that conventional programs alone often fail to bridge. However, patients also reported technology-related challenges. Based on the outcomes and qualitative review from this study, refinement in EMG-based technology, including hardware and software for video games, is a requisite for future use in clinical trials or other clinical applications. Despite the technical challenges reported, patients were able to complete a rigorous protocol of lower-body rehabilitation exercises. The successful completion of the study protocol indicated that an EMG-based video game intervention can be delivered both in-person and remotely.

Study limitations and strengths

The first limitation is that participants were not blinded; they knew what type of treatment they were receiving. This was mitigated by having the assessor group blinded. Second, the study had a smaller sample size than planned; therefore, it may be underpowered for detecting between-group differences. Third, both treatments were performed under supervision. Therefore, the results might not represent true compliance and adherence when patients undergo such training alone at home. Fourth, a controlled trial using real placebo video games is indicated in the future. It may be performed by changing the games with cognition and attention stimulation in a sitting posture. Fifth, we followed the patients for only 10 weeks. Originally, this study was planned for 12 weeks of intervention; however, due to time loss during the COVID-19 lockdown, the study protocol was modified to 10 weeks. Therefore, a trial comparing therapeutic exercise and active video games using a home-based design with a long-term follow-up is needed.

A further limitation concerns the generalizability of the current findings. The trial was conducted exclusively in an English-speaking population using English-language game interfaces and outcome measures, limiting applicability to non-English-speaking patients for whom language barriers may compound existing technology usability challenges. Additionally, the sample comprised participants with mild to moderate knee OA, and findings may not extend to those with more severe disease, who typically present with greater pain, functional limitation, and lower exercise tolerance, all of which could attenuate the motivational and adherence benefits observed in the current cohort. Future trials should incorporate multilingual interface options and recruit across the full spectrum of OA severity to establish the broader clinical applicability of KneeBRIGHT.

Lastly, the motivational benefits reported by KneeBRIGHT participants in the qualitative findings may partly reflect novelty-driven engagement rather than durable intrinsic motivation, and future trials incorporating longer follow-up periods and repeated engagement measures at multiple timepoints would help distinguish genuine adherence benefits from transient novelty effects. With the limitations listed, one of the major strengths of this study was that it indicated that it is possible to deliver the physical therapy intervention in a hybrid telemedicine format. Patients were given the device to use for exercises. Therefore, it is clearly possible to prescribe and deliver the video game-based intervention using the telemedicine platform.

## Conclusions

Ten weeks of rehabilitation exercises administered with a combination of in-person, supervised sessions and at-home sessions was effective in improving strength and perceived knee joint function, but not balance in patients with knee OA. Quadriceps strength showed positive trends in both groups; however, these did not reach statistical significance, and no significant between-group differences were observed. These findings were broadly consistent between patients who completed standard rehabilitation and those in the KneeBRIGHT group, with covariate-adjusted between-group comparisons revealing no statistically significant differences across KOOS subscales or IKDC scores. Additionally, qualitative findings indicated that engagement with the KneeBRIGHT system was associated with patient-reported enjoyment, motivation, and perceived accountability during exercise. Taken together, the results suggest that EMG-biofeedback-based video games deliver rehabilitation outcomes comparable to standard exercise for perceived knee function, without demonstrating superiority on clinical outcome measures, while offering potential advantages in patient motivation and engagement that warrant further investigation in larger, adequately powered trials.

## Appendices

Appendix 1 

**Table 5:** Supplementary information for video games

Parameter	Detail
EMG signal filtering	Bandpass filter range (6.26-250 Hz)
Normalization procedure	Signal normalized to maximum voluntary isometric contraction (MVIC) collected at baseline
Sampling rate	2000 Hz
Signal processing	Root mean square (RMS) envelope with 300 ms moving window
Game activation threshold	EMG activation threshold set at 40% MVIC for standard difficulty, adjustable via software settings
Progression rules	Rehabilitation progressions were pre-built into the game levels based on established rehabilitation guidelines, with the option for manual adjustments to exercise intensity (through activation thresholds) by the treating physical therapist

Appendix 2 

**Table 6:** Key themes and subthemes

Themes/subthemes	Categories	Quote #	Participant quotes (participant number)
The KneeBRIGHT games for exercise are motivating because they are fun and keep you accountable			
	Games are enjoyable and fun	1	I was initially really intrigued by [games] it. For the most part, I really enjoyed it. I enjoyed the engagement, and I was motivated by the little bells and whistles that allowed me to get extra points. For the most part, I enjoyed the games themselves. I liked just watching the scenery, and being a part of something that was just having to lift my legs or do squats. (P1)
		2	I thought it was fun. It made it more fun than just doing a bunch of exercises, which I have done before. I have been to PT and I had to do stuff at home and it was just kind of boring. (P7)
		3	[The game exercise program] has specific timing and all of those things. If the instructions are clear, I think it’s great. I enjoy doing. I’m appreciative that I’m in the treatment group because if I hadn’t been I think that it would be really boring. If I had surgery and was rehabilitating, I would love to have something like that. (P25)
		4	I liked that I was engaged and concentrating on what I was doing. It really held my attention to do what needed to be done. If there was noise in the background, it kept my attention. I liked that part of it. (P25)
		5	I enjoyed using the computer game for the 10 weeks that I used it. I think that it was beneficial. (P31)
		6	I liked the idea of being in a balloon. Sort of got into the story of delivering the packages. The whole rescue thing. The kayak, I liked the idea of being on the water and being able to steer a boat through different places. Through the targets and by flexing my quadriceps muscles and things like that. I enjoyed that. (P35)
	Seeing muscle activation feedback keeps you accountable	7	I see it as monitoring the muscles that I want to fire and telling me how well I’m doing. The video game gave me this drive to do this because the computer would rat on me if I cheated, just like my Fitbit. I try to get 10000 steps a day, and I’m addicted to it. It’s the same idea. (P28)
		8	The thing that I like about it is that it counts for you, and I hate repetitious counting, so it does that. And it gives you cues and runs you through it. It seemed to me like it took less time [than regular exercise]. It didn’t seem like much of a time commitment. In fact, I didn’t even know how long the sessions were, but they went quickly. And I hate machines too. (P28)
		9	I think the thing I liked the most was that you could see the green bar and you could tell when you [how hard you were working]. (P28)
		10	I liked the fact the with the electrodes on my legs I could see what was really going on in my muscles, so that I could judge the effort that I was putting forth. (P31)
		11	I thought it was beneficial… I appreciate that you could see the red-green or above the line [activation level], that was a driving force for me. (P37)
The exercises conducted with the KneeBRIGHT games are an effective alternative to regular exercise			
	Noticeable improvements from the game exercises	12	All of the exercises made me feel like I was getting stronger, which was great. (P1)
		13	It seemed pretty comprehensive. I’ve been to PT for a long time, and what was included in this program were a lot of the same kinds of things that I had been doing at PT. So it was a good routine. (P7)
		14	I’ve had a gradual improvement in my knee... There was a time where I had pain and sharp pains in my knee for quite a while. Over the course of the last 6 months that’s improved quite a bit. I don’t have the sharp pains anymore. (P14)
		15	I’m sure that I’m better off than I was before. I feel like my legs are stronger… I feel better. I feel good. (P25)
		16	Overall, the exercising was good. I believe my knee is stronger. I think I’ve felt it in walking up and down stairs, has improved. That’s kind of my overall. (P26)
		17	I feel like I really did benefit from this... I did not understand that your body compensates and protects a bad joint which actually makes it worse. I just did not know that. Now that I know that I can compensate for that by actually [correctly] doing the exercise. (P28)
	Patients appreciate the convenience and structure of the games	18	I think that [video game exercise sessions] would be better [than PT] because you don’t have to drive somewhere to do it. (P14)
		19	[Video game exercises] are great. It provides structure. (P21)
Patients would like to see additional encouragement regarding their performance and progress			
		20	I know I needed support from {the study PT} to make sure all my body positions were correct, if I had just kept doing the exercises in the video the way I was doing them, I probably wouldn't have gotten as much out of them. (P4)
		21	When you finished a session, it would give you [a summary] about how many things [game resources] you got, but there was nothing that really gave me a summary of how I was doing or how I was progressing. And I understand that this is a research study, so it’s got to be limited. Eventually it would be nice to have positive feedback. Some testing similar to what you’ve got going on here that shows progression. If you’re going to continue doing this, you’d like to know you’re getting something out of it. (P26)
		22	I didn’t get a lot of feedback to reward myself like “atta boy” or “pat on the back” or a graphic display of “it looks like you’re increasing your strength here look this graph is bigger than it was a month ago.” There was nothing rewarding after going through the exercise a few times. There was no sense of achievement. (P6)
		23	Because it’s not really giving you and feedback or encouragement above and beyond the same thing that it’s done for the entire period of time, it does not seem to build incentive to continue or to Improve. If there was any kind of tabulation at the end, say “that’s great, you have completed this many sessions of this particular exercise.” Even just a little nugget like that to say that’s great, you’ve achieved this or you’ve done this. And that at least is some kind of reward for doing it. (P6)
Patient thoughts on the hardware and software usability of the system			
	Hardware issues are frustrating	24	It was a little disappointing when I couldn’t do the exercise [due to technical difficulties], so I think that one really big thing to change about those is to make them much more reliable than what my experience was. I understand that I was in a research study and its ok because that what you all were learning how to do. I don’t really have any big changes. It’s most just making the system more reliable. (P1)
		25	The only thing I really didn’t like was that I had a lot of trouble with the transmitters. (P21)
	Needed improvement in graphics and timing	26	The game was quite cumbersome, in that there were portions of the game that were obscured. When the balloon is there or sometimes the targets that you were looking for were obscured because they were under other items. So, you really have to snoop around to find them and by the time you find them they’re almost difficult to get to in time to whatever the reward would be to go through the ring before you do the exercise. If that was something to side that would be one thing, but it’s straight overhead and its under other stuff so you can’t really get to it. (P6)
		27	There wasn’t transition time, for instance the knee resistance with the band and getting out of the band to do either the step up or whatever came after the resistance band, so there needed to be some transition time. (P7)
		28	The sensitivity when moving things didn’t seem to be consistent between the different video games. Some of them you could move and control and line up and kind of nail. Others you’d be coming over and all of a sudden it was out of range, and you couldn’t get back. Some kind of a better scaling of the rate at which things occur would probably have been better... There were several places where the little target you had to go through was almost invisible. It was really hard to see. In all of them, it seemed like when it would pop up the screen to get ready for the next exercise, it would still be moving. So, some of your targets would be behind the little “get ready for this” and sometimes you’d hit them and sometimes you wouldn’t. It really shouldn’t be targets there. (P26)
		29	There were times where I wasn’t really sure was going to come next. Sometimes there wasn’t enough time to get from one position to another. I found that inconvenient. (P31)
		30	I would improve the graphics. Make them more consistent from one game to the other. Like on the balloon, it responded perfectly. You move it to the left it, it goes to the left. On others like the horse and the bicycle, not so much. I think that improving the graphics, making them more consistent from one game to the next. (P35)
